# Abdominal aortic aneurysm progression: A review of preclinical and clinical data

**DOI:** 10.1007/s00392-025-02764-8

**Published:** 2025-11-10

**Authors:** Nadjib Schahab, Sara Würbel, Lucas Busch, Georg Nickenig

**Affiliations:** 1 Heart Center Bonn, Department of Medicine II, University Hospital Bonn, Bonn, Germany; 2 Division of Cardiology, Pulmonology and Vascular Medicine, Medical Faculty, Heinrich-Heine University, Düsseldorf, Germany

**Keywords:** Abdominal aortic aneurysm, Screening, Aneurysm rupture, Aortic disease, Aortic aneurysm pathophysiology

## Abstract

Despite advancements in surgical and endovascular interventions and improved screening protocols, abdominal aortic aneurysm (AAA) remains a progressive vascular condition associated with significant morbidity and mortality owing to rupture. However, the mechanisms underlying the progression of AAA are poorly understood. AAA progression is driven by complex biological mechanisms, including endothelial dysfunction, chronic inflammation, extracellular matrix (ECM) degradation, proteolytic activity, and vessel wall remodeling. Some studies highlight proteases like matrix metalloproteinases in ECM remodeling, while others focus on miRNAs regulating inflammation; risk factors such as smoking and hypertension further increase vascular wall stress and aneurysm growth. The lack of detailed understanding limits the development of targeted therapies and individual risk assessments. Risk-prediction models are promising; however these models require further external validation to ensure reliability and clinical applicability. Personalized approaches integrating biomechanics and advanced imaging may improve rupture risk assessment. However, trials of antibiotics and renin–angiotensin system inhibitors have shown limited benefit. Observational studies have suggested potential benefits of metformin and statins. Preclinical studies have proposed that targeting inflammatory pathways such as the NOD-like receptor P3 inflammasome is a novel therapeutic strategy to mitigate aneurysm progression. Furthermore, innovative nanoparticle-based drug delivery systems have been explored to deliver matrix metalloproteinase inhibitors directly to the aneurysm site to prevent aneurysm expansion while minimizing systemic side effects. Integrative research is urgently needed to clarify AAA progression, improve outcomes, and enable personalized detection of high-risk subthreshold AAAs while avoiding overtreatment of low-risk cases. This review consolidates current knowledge on AAA pathophysiology, epidemiology, and treatment challenges.

**Graphical Abstract:**

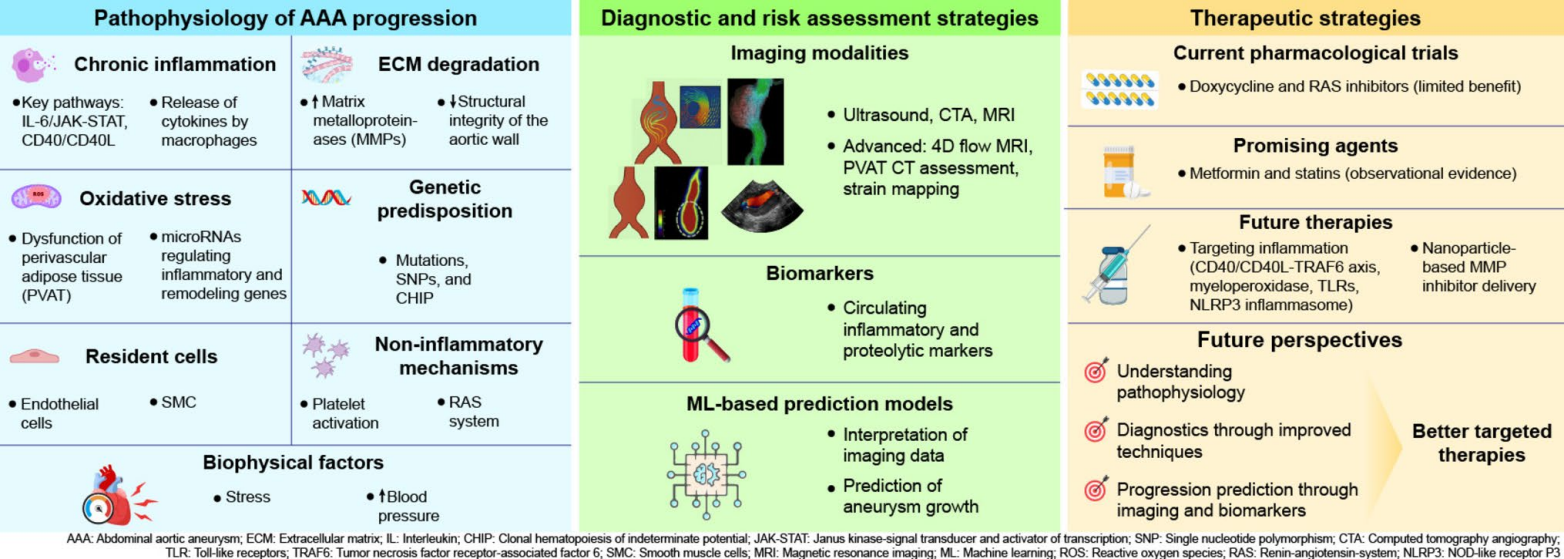

## Introduction

An abdominal aortic aneurysm (AAA) is a localized dilatation of the abdominal aorta to a diameter ≥ 30 mm or 50% larger than the normal abdominal aortic diameter [1–3]. AAA can also be defined as the maximum infrarenal aortic diameter ≥ 1.5 times the expected normal infrarenal or suprarenal aortic diameter [4]. This relative increase in diameter is beneficial for defining AAA in women, who generally have smaller mean aortic diameters than men [5, 6].

AAA are typically asymptomatic until rupture, which is a catastrophic event with a mortality rate exceeding 80% in the absence of surgical or endovascular interventions [1–3, 5]. In 2017, AAA accounted for approximately 167,200 deaths and 3 million disability-adjusted life years globally. These figures may underestimate the true burden due to the limitations of postmortem diagnostics [7, 8].

The etiology of AAA development and progression remains unclear. Endogenous and exogenous risk factors, including genetic variants, age, smoking, hypertension, and hypercholesterolemia, have been identified as predisposing factors. At the cellular and molecular levels, inflammatory cascades mediated by circulating and resident immunocompetent cells and dysfunction within the vascular wall, including the endothelium, vascular smooth muscle cells (VSMCs), and extracellular matrix (ECM), are thought to play critical roles. Within this pathological network, noncoding RNAs, specific cytokines, growth factors, and proteases initiate AAA development and accelerate its progression. [1, 7, 9, 10]

In addition to these essential cellular alterations, biophysical factors including pulse wave propagation, wall stress, wall stiffness, and vascular diameter significantly influence the progression [11]. Despite recent scientific advancements, the molecular mechanisms underlying abdominal aortic disease remain unclear. This gap contributes to the lack of efficient early diagnostic tools, absence of causative treatment strategies, and inability to prevent disease progression and associated clinical events.

Screening programs targeting men aged ≥ 65 years have demonstrated reductions in aneurysm-related mortality. However, its effect on all-cause mortality remains debated [4, 12–21]. Emerging advances in imaging and biomarker discovery may provide promising avenues for early detection and risk stratification [8, 9, 22, 23].

## Epidemiology

AAA affects approximately 2–8% of men aged ≥ 65 years in industrialized nations, with a lower prevalence among women and younger populations. Advanced age was the strongest non-modifiable risk factor, with AAA prevalence peaking in men aged 75–84 years. Smoking is the most impactful modifiable risk factor, and hypertension exacerbates this risk in both sexes. Although clinical trials of antihypertensive therapy have not consistently slowed aneurysm expansion, large-scale epidemiological cohorts demonstrate that hypertension is a major determinant of AAA development. In the Tromsø Study, hypertension was associated with incident AAA (OR 1.54, 95% CI 1.03–2.30) [7]. The recent UK Biobank analysis of 397,019 participants confirmed that elevated diastolic blood pressure, isolated diastolic hypertension, and combined hypertension were significantly associated with aneurysm incidence, while systolic blood pressure alone was not [24]. Mendelian randomization analyses clearly support hypertension as a causal factor for AAA development and rupture. Notably, diastolic rather than systolic blood pressure appears to exert the predominant influence on aneurysm risk, underscoring the importance of rigorous blood pressure control [25]. A family history of AAA doubles the likelihood of developing this condition, emphasizing the importance of genetic susceptibility. Public health initiatives, particularly smoking cessation campaigns, have contributed to the decline in the incidence of AAAs in recent decades. However, geographical disparities persist; prevalence rates exceed 3% in Europe and the Western Pacific but remain below 1% in Asia. [5, 10, 15, 23, 26–32]

Among women, AAA is less common but becomes significant at an advanced age, particularly among those with hypertension, history of premature menopause, and smoking [12, 27, 29, 30, 33].

According to national screening studies in Germany, the prevalence of AAA among men aged > 65 years was estimated to be between 1.5% and 3.6%. Data from a retrospective study conducted in Germany between 2005 and 2014 showed that the incidence of non-ruptured AAAs (nrAAAs) increased by 16% in men and 42% in women. The incidence of ruptured AAAs (rAAAs) decreased by 30% in both sexes. During this time, in-hospital mortality rates were 3.3% for non-ruptured AAAs (nrAAAs) in men and 5.3% in women, and 39% for rAAAs in men and 48% in women. The overall aneurysm mortality rate in Germany has declined from 4.3 to 2.8 per 100,000 individuals. [34, 35]

The adoption of endovascular aneurysm repair (EVAR) has increased significantly. By 2014, 75% of nrAAAs and 36% of rAAAs were treated using EVAR, contributing to shorter hospital stays and improved survival rates. Approximately 12,000 invasive procedures are performed annually in Germany to manage intact and ruptured AAAs, reflecting the significant healthcare burden associated with this condition. [34, 35]

These trends highlight the improvements in AAA management, including the increased use of minimally invasive treatments and reduced mortality rates.

## Pathogenesis of AAA

### General biology and pathology

The aorta plays a central role in systemic circulation by conducting blood flow from the left ventricle and maintaining diastolic pressure through an elastic recoil. It ensures organ perfusion by dynamically regulating vascular resistance [36]. Aging and pathological processes lead to structural and functional remodeling of the aortic wall, reducing its elasticity and compliance [37, 38].

**Fig. 1:**
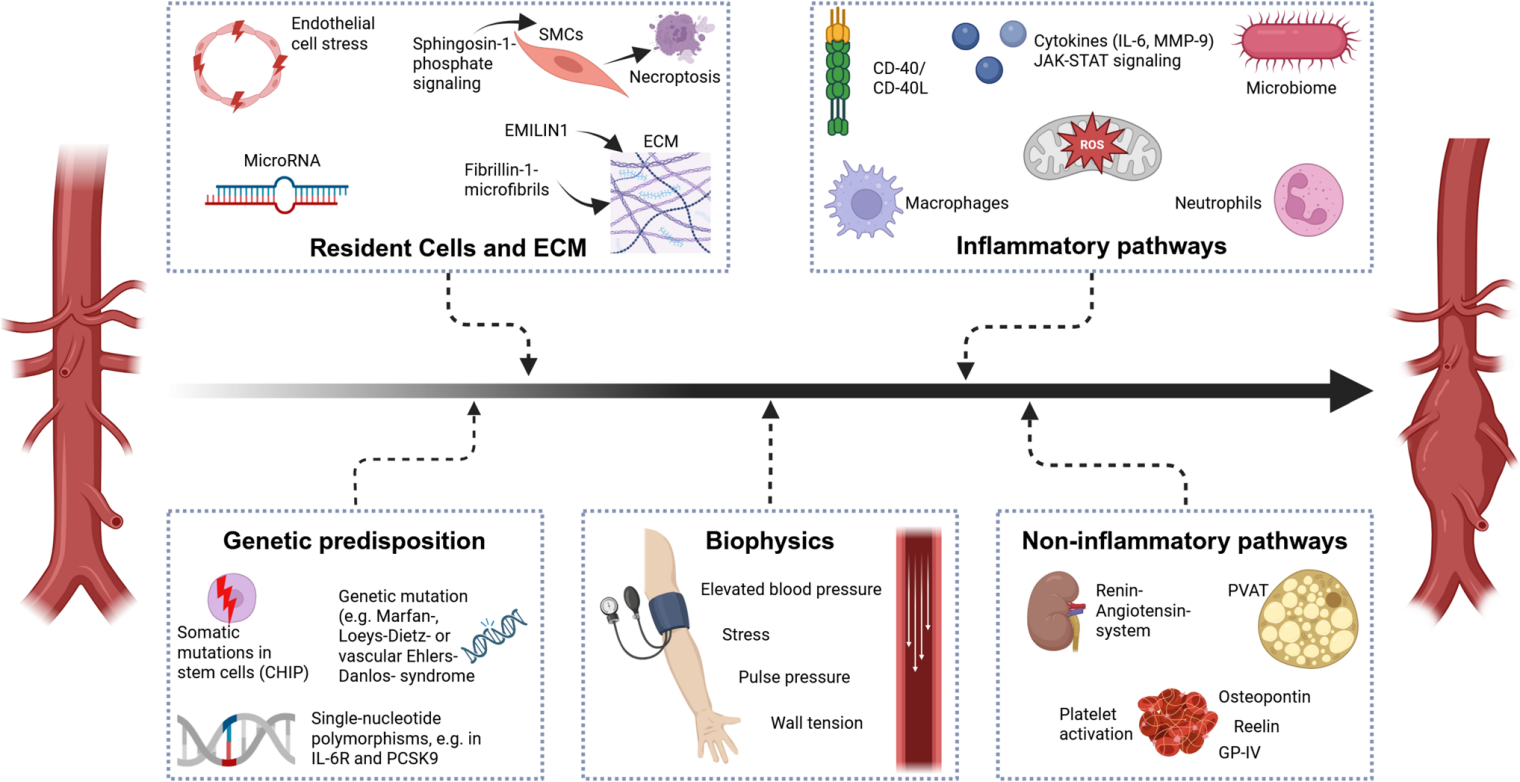
Schematic overview of key mechanisms involved in the pathogenesis of aortic aneurysms The figure summarizes extrinsic and intrinsic risk factors and molecular contributors to aortic aneurysm development. CD40/CD40L: Cluster of Differentiation 40 and its ligand; CHIP: clonal hematopoiesis of indeterminate potential; ECM: extracellular matrix; EMILIN-1: elastin microfibril interface-located protein 1; FBN1: fibrillin 1 gene; GPVI: glycoprotein VI; IL-6: interleukin 6; JAK-STAT: Janus kinase/signal transducer and activator of transcription; MMP-9: matrix metalloproteinase 9; PCSK9: proprotein convertase subtilisin/kexin type 9; PVAT: perivascular adipose tissue; SMCs: smooth muscle cells; SNPs: single-nucleotide polymorphisms; S1P: sphingosine-1-phosphate

Aneurysm formation is driven primarily by cumulative endothelial damage, cellular degeneration, inflammatory processes, biomechanical stress, and genetic predisposition. In addition, the interplay between extrinsic and intrinsic risk factors, along with infiltrating effector cells, acts synergistically to promote the complex pathogenesis of aortic disease (Fig. 1). [1, 2, 39, 40]

### Inflammatory pathways

Chronic inflammation is a hallmark of AAA. Infiltrating macrophages and neutrophils release proinflammatory cytokines (e.g., IL-6), T cells, particularly Th1 cells, promoting cytokine release (e.g. IFN-γ) and matrix metalloproteinases (MMP) (e.g., MMP-9), leading to ECM degradation [41]. Several studies have shown that the JAK-STAT signaling pathway, particularly via JAK2/STAT3 activation, plays a key role in AAA development. Xiao et al. demonstrated that blocking this pathway significantly reduces aneurysm growth in experimental models by lowering inflammatory cytokine expression and macrophage infiltration [42]. Genomic analyses further revealed that enhanced IL-6 signaling leads to the overactivation of JAK-STAT in AAA tissue, promoting vascular inflammation, smooth muscle cell (SMC) apoptosis, and extracellular matrix degradation [42, 43].

The CD40/CD40L co-stimulatory pathway has also been identified as a key mediator of aneurysm development. Kusters et al. showed that CD40 ligand (CD40L) deficiency protects against aneurysm formation, underlining the critical role of immune signaling in aneurysm pathogenesis [44].

Reactive oxygen species (ROS) are elevated in AAA tissues [41]. Yang et al. further emphasized the role of ROS and dysfunctional mitochondria in promoting MMP activity and inflammatory gene expression. These oxidative processes are exacerbated by the simultaneous downregulation of antioxidant systems such as superoxide dismutase and catalase in aneurysmal tissues [40]. Gao and Guo explored the role of hypoxia, particularly its association with intraluminal thrombi (ILT) and vasa vasorum dysfunction. ILT impairs oxygen diffusion into the aortic wall, resulting in local hypoxia, which induces the expression of Hypoxia-Inducible Factor 1-alpha (HIF-1α) and promotes the formation of neovessels from the adventitia. These newly formed microvessels are often fragile and serve as entry points for inflammatory cells, further accelerating wall degradation [41]. Emerging evidence highlights the gut microbiome as a critical factor in AAA development, with dysbiosis linked to heightened inflammation, oxidative stress, and arterial remodeling. Notably, gut-derived metabolites and bacteria appear to modulate local renin-angiotensin system (RAS) activity, further influencing aneurysm formation (Fig. 1) [40].

### Non-inflammatory pathways

In addition to inflammation, patients with AAA frequently exhibit elevated platelet activity. Wagenhäuser et al. showed that platelet depletion mitigated aneurysm formation and vascular remodeling in experimental models. Platelets interact with immune cells, VSMCs, and ECM components, with osteopontin (OPN) acting as a key regulatory factor [45]. The glycoprotein VI receptor and its modulator reelin have also been implicated in these processes [46].

Perivascular adipose tissue (PVAT) is functionally comparable to brown adipose tissue (BAT) and contributes to vascular hemostasis and stiffness regulation. Recent studies have shown that the loss or dysfunction of PVAT, often due to aging or oxidative stress, contributes to increased arterial stiffness. Gao and Guo highlight the emerging concept of "outside-in" inflammation, where dysfunctional PVAT contributes to AAA pathogenesis by secreting proinflammatory adipokines and facilitating immune cell recruitment. The adipose tissue then becomes inflamed and fibrotic, amplifying the local inflammatory environment surrounding the aortic wall. [41, 47–49]

In parallel, the RAS plays a key role in non-inflammatory AAA progression, with the ACE/AngII/AT1R axis promoting oxidative stress and vascular smooth muscle cell dysfunction, while the protective ACE2/Ang-(1–7)/Mas receptor axis is downregulated in the aneurysmal tissue (Fig. 1) [40].

### Role of resident cells

The aortic wall exhibits region-specific cellular phenotypes, particularly among the endothelial and adventitial cells, which may contribute to the formation of site-specific aneurysm formation [50]. MicroRNAs, including miR-29b and miR-24, regulate vascular inflammation and remodeling and may serve as potential diagnostic or prognostic biomarkers. Toyama et al. demonstrated that elevated levels of exosomal miR-501-3p promoted vascular stiffness, suggesting its potential as a biomarker and therapeutic target for vascular remodeling disorders (Fig. 1) [51].

Building on the role of resident cells, Cho et al. used single-cell transcriptomics to identify distinct SMC subpopulations in aneurysmal tissues, including inflammatory and modulated phenotypes, with low contractile marker expression and high proteolytic activity. These cell states, which are regulated by transcription factors such as KLF4, MYOCD, and TCF21, contribute to medial degeneration. In addition, the fragmentation of elastin contractile units, which anchor elastin fibers to VSMCs, has been implicated as an early structural defect preceding overt wall dilatation [52].

### Role of ECM

The ECM is fundamental to the mechanical stability of the aortic wall and regulates cellular signaling.

Fibrillin-1 microfibrils and ECM proteins such as EMILIN1 are essential for maintaining elastic properties and tensile strength. Mutations or deficiencies in these proteins disrupt the ECM integrity and promote aneurysm development. Sphingosine-1-phosphate signaling, which modulates SMC phenotype and inflammatory responses, has been associated with increased susceptibility to aneurysm rupture. [53–55]

Several studies have reported an imbalance between extracellular matrix (ECM) degradation and synthesis in aneurysmal tissue, marked by elevated MMP and ADAMTS activity, along with reduced lysyl oxidase (LOX) expression, ultimately weakening the structural integrity of the aortic wall [56, 57]. Cho et al. further highlight the relevance of disrupted elastin–SMC interactions and localized matrix fragmentation (Fig. 1) [52].

### Biomechanical forces

Advanced imaging techniques, such as magnetic resonance imaging (MRI) and fluorine-19 cardiovascular MRI (19F CMR), provide insight into the underlying mechanisms of AAA progression by revealing flow disturbances and vascular inflammation, both of which contribute causally to aneurysm expansion and rupture. Recent findings by Mutlu et al. underscored the role of altered blood flow patterns in the biomechanics of aneurysms. Using computational fluid dynamics analysis, this study demonstrated that regions within AAAs exhibiting low time-averaged wall shear stress and a high oscillatory shear index were associated with increased mechanical stress and a higher risk of rupture (Fig. 1) [58].

### Genetic predisposition

Genetic factors are major contributors to AAA pathogenesis. Genome-wide association studies (GWAS) have identified single-nucleotide polymorphisms in genes such as IL-6R and PCSK9, which are involved in inflammatory signaling and lipid metabolism, and therefore play a role in the development of AAA [1, 59]. Furthermore, GWAS and Mendelian randomization analyses demonstrate an overlap between blood pressure–associated loci and AAA. In particular, genetically elevated diastolic blood pressure increases aneurysm risk, thereby supporting a shared genetic architecture [25].

Somatic mutations in hematopoietic stem cells, known as clonal hematopoiesis of indeterminate potential (CHIP), have emerged as novel risk factors for vascular inflammation and AAA development. CHIP-associated mutations, particularly in genes such as *TET2* and *JAK2*, promote proinflammatory phenotypes in immune cells, accelerating matrix degradation and aneurysm progression [60–62]. Cho et al. also emphasized the role of monogenic connective tissue disorders such as Marfan syndrome (FBN1), Loeys–Dietz syndrome (TGFBR1/2, SMAD3/4), and vascular Ehlers–Danlos syndrome (COL3A1) in predisposing patients to aneurysm formation. These mutations impair ECM integrity and dysregulate TGF-β signaling. While TGF-β typically promotes VSMC stability and matrix synthesis, its aberrant activation has been implicated in pathological wall remodeling (Fig. 1) [52].

## Diagnosis and screening

AAAs are often asymptomatic and are diagnosed incidentally during imaging for unrelated reasons. Standard imaging modalities include ultrasonography (US), computed tomography angiography (CTA), and cardiovascular magnetic resonance imaging (CMR), all of which play important roles in detection, monitoring, and preoperative planning [5, 63, 64].

The US remains the gold standard for AAA screening primarily because of its accessibility and cost-effectiveness. However, significant inter-observer variability and methodological inconsistencies exist across studies. Schäberle emphasized the importance of orthogonal-axis-independent measurements using the leading-edge-to-leading-edge method, noting that discrepancies of up to 5 mm can occur depending on the measurement technique, potentially leading to misclassification or overtreatment [65]. CTA provides excellent anatomical resolution and is invaluable for preoperative planning despite associated radiation and contrast exposure [63, 64]. Meyrignac et al. combined volumetric assessment and wall shear stress analysis derived from CT imaging to evaluate the risk of AAA progression. Their study showed that combining morphological and hemodynamic data improves the prediction of aneurysm growth and rupture risk, highlighting the importance of a comprehensive biomechanical approach to AAA management [66].

Ginzburg et al. showed that CT-based analysis of perivascular adipose tissue (PVAT) using AI-assisted segmentation can detect localized inflammatory changes in patients with abdominal aortic aneurysms (AAA) by measuring the increased PVAT density near the aneurysmal wall. These findings suggest that PVAT attenuation may serve as a non-invasive imaging biomarker for vascular inflammation and AAA progression [49].

CMR is an alternative for patients with contraindications and offers reliable wall and lumen assessments without ionizing radiation [67]. Bouvain et al. demonstrated that 19F MRI allows the visualization of localized inflammatory “hot spots” in cardiovascular tissues, offering valuable diagnostic precision. Furthermore, they showed that multi-parametric MRI provides a comprehensive, non-invasive assessment of flow dynamics and immune cell recruitment in abdominal aortic pathologies, thereby surpassing the limitations of conventional diameter-based evaluations [68].

The maximum anteroposterior diameter is the cornerstone of AAA diagnosis and treatment decision-making. Although prevalence is lower in women, affected individuals often exhibit more rapid expansion and higher rupture risk at smaller diameters. Consistent with these differences, current European Society for Vascular Surgery (ESVS) guidelines and the 2024 ESC Guidelines for the Management of Peripheral Arterial and Aortic Diseases recommend a lower diameter threshold for elective repair in women. A threshold of 5.5 cm in men and 5.0 cm in women is widely accepted as an indication for the elective repair of AAA, as supported by randomized trials and guidelines [63, 64, 69, 70]. However, this metric may not reflect the true aneurysm burden or risk profile of irregular or elongated aneurysms. A meta-analysis by Kandail et al. suggested that volumetric measurements are superior for tracking growth and assessing rupture risk [71].

The Copenhagen Aneurysms Cohort (COACH) is a prospective cohort study focusing on patients with AAAs. A key aspect of the COACH study was the development and application of advanced US techniques, including three-dimensional ultrasound (3D-US), to accurately measure aneurysm volume and analyze growth rates. A study within the COACH cohort demonstrated that volume measurement using 3D-US was more sensitive than traditional diameter measurements for detecting growth changes [72]. Another research focus within the COACH study examined the biomechanical properties of the aortic wall in patients with AAA. Ultrasound was used to analyze local strain patterns of the aortic wall, providing insights into potential risk factors for aneurysm growth and rupture. These findings suggest that strain pattern analysis is a promising method for improving AAA risk assessment [73]. Although aneurysm length is not commonly used to determine intervention thresholds, it is relevant to procedural planning, particularly in the context of EVAR [64].

The 2024 ESC Guidelines for the Management of Peripheral Arterial and Aortic Diseases recommend one-time duplex ultrasound (DUS) screening for AAA in men aged > 65 years who smoke (Class I, Level A). Additionally, opportunistic screening is suggested for patients with peripheral arterial disease undergoing DUS and for men over 65 years and women over 75 years of age undergoing echocardiography, even in the absence of risk factors (Class IIa, Level B). In patients with AAA, the ESC guidelines further recommend that femoro-popliteal aneurysm screening with DUS should be considered (Class IIa, Level C), as such aneurysms are present in up to 10–20% of cases. These recommendations are consistent with the 2024 ESVS Guidelines on AAA management, which likewise emphasize the importance of screening for concomitant peripheral aneurysms, particularly femoro-popliteal aneurysms, in patients with AAA. [63, 64].

The Multicenter Aneurysm Screening Study (MASS) included 67,800 men aged 65–74 years and demonstrated a 42% reduction in AAA-related mortality after four years [20]. However, a 15-year follow-up revealed that this benefit diminished over time, with only an 11% reduction that was not statistically significant (HR 0.89, 95% CI 0.60–1.32), raising questions regarding the long-term effectiveness of one-time screening [13]. The loss of benefit was attributed to factors such as non-attendance, comorbidities precluding surgery, and the development of new aneurysms later in life [14].

Similarly, the Western Australia Trial found that while population-wide screening did not reduce overall AAA mortality, a significant mortality reduction was observed in the 65–74-year subgroup [16]. The Danish Cardiovascular Screening (DANCAVAS) trial, which included cardiovascular screening in over 46,000 men, showed a modest reduction in overall mortality (HR, 0.95) and a significant reduction in stroke incidence, but no significant AAA-specific mortality reduction after 5 years [74]. In Germany, a nationwide AAA screening program was introduced in 2018, offering a one-time US examination for all men aged 65 years through statutory health insurance (GKV) reimbursements. This measure aligns with the European guideline recommendations and is primarily based on evidence from large international studies [63]. However, German data regarding the effectiveness of this screening program remain limited. A recent analysis of nationwide hospital discharge data from 2005 to 2021 showed that hospitalizations for ruptured and non-ruptured AAAs increased by 14% between 2005 and 2019 but subsequently declined during the COVID-19 pandemic, likely because of reduced healthcare utilization. The same study noted a clear procedural shift towards endovascular repair (EVAR), which is consistent with international trends [75].

### Prevention of AAA progression

Small AAAs, defined as < 5.5 cm in men and < 5.0 cm in women, are generally managed conservatively through regular imaging surveillance and rigorous control of cardiovascular risk factors [12, 63, 64, 76, 77]. Despite intense research efforts, pharmacological strategies have consistently failed to demonstrate reliable clinical benefits for slowing aneurysm growth.

Randomized trials evaluating antibiotics and agents targeting inflammation or protease activity, such as doxycycline [30] and roxithromycin [78, 79], have yielded conflicting or negative results. Similarly, renin–angiotensin system inhibitors, including perindopril [80] and telmisartan [81], did not reduce aneurysm growth despite achieving blood pressure reduction (Table 1) [23, 26, 63, 82–89].

**Table 1:** Selected RCTs Evaluating Therapeutic Interventions in Abdominal Aortic Aneurysm

Randomized Controlled Trials on Treatment of AAA								
First Author et al	Year	Title of the Study	Mechanisms Reviewed	Outcomes	Categorization	Sample Size (*n*)	Study Type	Key Findings
Mosorin et al	2001	Use of doxycycline to decrease the growth rate of abdominal aortic aneurysms: A randomized, double-blind, placebo-controlled pilot study	Imaging: Ultrasound measurement of AAA diameter. Biomarkers: Chlamydia pneumoniae IgG and IgA antibody titers, C-reactive protein (CRP) levels	Primary: AAA expansion rate. Secondary: Number of AAA ruptures or repairs, C. pneumoniae antibody titers, serum CRP concentration	Therapeutic trial (doxycycline vs. placebo for reducing AAA growth)	32 randomized patients (doxycycline *n* = 17, placebo *n* = 15)	Randomized, placebo-controlled, double-blind, single-center pilot study	Doxycycline significantly reduced AAA expansion compared with placebo during the 6–12-month (p = 0.01) and 12–18-month periods (p = 0.01). Overall, fewer doxycycline patients showed significant AAA expansion (≥ 5 mm). Higher expansion rate correlated with higher IgG antibody titers in the placebo group (p = 0.03). CRP levels significantly decreased after doxycycline treatment (p = 0.01). Results suggest potential benefit of doxycycline in AAA growth management, but larger studies required. However, because of the small size of this randomized study and of the potentially confounding effect of pretreatment risk factors, doxycycline-based treatment cannot be justified only on the ground of the current results
Vammen et al	2001	Randomized double-blind controlled trial of roxithromycin for prevention of abdominal aortic aneurysm expansion	Imaging: Ultrasound measurement of AAA diameter. No specific biomarkers mentioned	Primary: Annual expansion rate of AAA diameter. Secondary: Referral for surgical repair, rupture, mortality	Therapeutic trial (roxithromycin vs. placebo for reducing AAA growth)	92 randomized patients (roxithromycin *n* = 43, placebo *n* = 49)	Randomized, placebo-controlled, double-blind clinical trial	Roxithromycin significantly reduced mean annual AAA expansion rate (1.56 mm vs. 2.75 mm; p = 0.02). Fewer patients in the roxithromycin group required surgery (9 vs. 22%, p = 0.05). Suggests a beneficial effect of roxithromycin on slowing AAA progression
Propranolol Aneurysm Trial Investigators (Laupacis et al.)	2002	Propranolol for small abdominal aortic aneurysms: Results of a randomized trial	Imaging: Ultrasound measurement of AAA diameter (anteroposterior and transverse); no specific biomarkers mentioned	Primary: Mean annual AAA growth rate (cm/year). Secondary: mortality, elective AAA surgery, medication withdrawal, quality of life (SF-36)	Therapeutic trial (propranolol vs. placebo for slowing AAA growth)	548 randomized patients (propranolol *n* = 276, placebo *n* = 272)	Randomized, placebo-controlled, double-blind, multicenter trial	No significant difference in AAA growth rate between propranolol (0.22 cm/year) and placebo (0.26 cm/year; p = 0.11). Higher withdrawal rate due to adverse effects with propranolol (42.4% vs. 26.8%, p = 0.0002). Slight trend toward fewer elective surgeries in propranolol group (20.3% vs. 26.5%; adjusted p = 0.03). Significantly poorer quality of life (physical functioning, physical role, vitality) with propranolol. Conclusion: Propranolol does not clinically significantly slow AAA growth and is poorly tolerated
Karlsson et al	2009	The effect of azithromycin and Chlamydophilia pneumonia infection on expansion of small abdominal aortic aneurysms—A prospective randomized double-blind trial	Imaging: Ultrasound measurement of AAA diameter (anteroposterior), CT scan for aneurysm volume calculation. Biomarkers: Serological markers (IgA and IgG) for Chlamydophilia pneumoniae (Cpn)	Primary: Annual AAA expansion rate. Secondary: Surgical intervention, aneurysm rupture, correlation of Cpn serology with AAA growth	Therapeutic trial (azithromycin vs. placebo for reducing AAA growth)	247 randomized patients (azithromycin *n* = 123, placebo *n* = 124)	Randomized, placebo-controlled, double-blind, multicenter trial	Azithromycin did not reduce AAA expansion rate compared with placebo (median growth rate 0.22 cm/year in both groups, p = 0.85). No correlation between Cpn serology and AAA growth. Patients treated with combined aspirin and statins had significantly reduced AAA expansion rates compared with those without these medications (0.14 cm/year vs. 0.27 cm/year, p < 0.001)
Høgh et al	2009	Intermittent Roxithromycin for Preventing Progression of Small Abdominal Aortic Aneurysms: Long-Term Results of a Small Clinical Trial	Imaging: Ultrasound measurements (anterior–posterior AAA diameter). No specific biomarkers mentioned	Primary: AAA growth rate and referral for surgery (AAA diameter > 50 mm)	Therapeutic trial (intermittent roxithromycin vs. placebo for reducing AAA growth and surgical referral)	84 randomized (roxithromycin *n* = 42, placebo *n* = 42)	Randomized, placebo-controlled trial (single-center, double-blind)	- Intermittent roxithromycin treatment significantly reduced mean annual AAA growth rate by 36%. Lowered risk of surgical referral by 57% after adjusting for confounders. Suggests a potential benefit of roxithromycin in slowing AAA progression; however, small sample, further larger trials needed
Meijer et al	2013	Doxycycline for Stabilization of Abdominal Aortic Aneurysms: A Randomized Trial	Imaging: Ultrasound (AAA diameter). No specific biomarkers mentioned	Primary: AAA growth rate at 18 months Secondary: AAA growth at 6 and 12 months, elective surgery, mortality	Therapeutic trial (doxycycline vs. placebo for reducing AAA growth)	286 randomized (doxycycline *n* = 144, placebo *n* = 142)	Randomized, placebo-controlled trial (multicenter, double-blind)	- Doxycycline did not reduce AAA growth; growth was slightly higher in doxycycline group (4.1 mm vs. 3.3 mm, placebo). No influence on elective AAA repair or time to repair. Higher adverse event withdrawal rate in doxycycline group. Concludes doxycycline not beneficial in slowing AAA progression
Sillesen et al	2015	Randomized clinical trial of mast cell inhibition in patients with a medium-sized abdominal aortic aneurysm	Imaging: Ultrasound measurement of AAA diameter (anteroposterior adventitia). Biomarkers: Plasma inflammatory biomarkers (e.g., tryptase)	Primary: Change in AAA diameter over 12 months. Secondary: AAA repair, rupture, cardiovascular complications, mortality, and biomarker levels	Therapeutic trial (pemirolast vs. placebo for reducing AAA growth)	326 randomized (placebo *n* = 84, pemirolast 10 mg *n* = 80, 25 mg *n* = 78, 40 mg *n* = 84)	Randomized, placebo-controlled, double-blind, multicenter trial	No significant difference in AAA growth between placebo and any dose of pemirolast. Overall mean growth rate was 2.42 mm/year. No effect on biomarkers, AAA repair, rupture, or cardiovascular outcomes. Pemirolast showed no clinical benefit in reducing growth rate of medium-sized
Bicknell et al	2016	An evaluation of the effect of an angiotensin-converting enzyme inhibitor on the growth rate of small abdominal aortic aneurysms: a randomized placebo-controlled trial (AARDVARK)	Imaging: Ultrasound measurements (external antero-posterior diameter, longitudinal plane). Biomarkers: Creatinine and electrolytes	Primary: Growth rate of small abdominal aortic aneurysms (AAA). Secondary: Blood pressure changes, time to reach an AAA diameter of 5.5 cm, elective surgical intervention, or AAA rupture	Therapeutic trial (comparison of perindopril, amlodipine, and placebo for reducing AAA growth rates)	224 randomized patients (placebo *n* = 79, perindopril *n* = 73, amlodipine *n* = 72)	Randomized, placebo-controlled trial (multicenter, single-blind)	- No significant difference in AAA growth rate between perindopril, amlodipine, and placebo groups - No significant difference in the number of patients whose AAA reached ≥ 5.5 cm or required surgery - Greater blood pressure reduction with perindopril did not translate into slower AAA growth
Pinchbeck et al	2018	Randomized Placebo-Controlled Trial Assessing the Effect of 24-Week Fenofibrate Therapy on Circulating Markers of Abdominal Aortic Aneurysm: Outcomes From the FAME-2 Trial	Biomarkers: Osteopontin, kallistatin, matrix metalloproteinase-9 (MMP-9), resistin, osteoprotegerin, D-dimer. Imaging: Ultrasound measurement of AAA diameter	Primary: Changes in serum osteopontin and kallistatin concentrations. Secondary: Changes in circulating concentrations of other AAA-associated biomarkers, AAA diameter	Therapeutic trial (fenofibrate vs. placebo for modification of AAA biomarkers and diameter growth)	140 randomized (fenofibrate *n* = 70, placebo *n* = 70)	Randomized, placebo-controlled, double-blind, multicenter	- No significant differences in serum osteopontin, kallistatin, or other AAA biomarkers between fenofibrate and placebo groups. No significant difference in AAA diameter growth between groups after 24 weeks. Fenofibrate significantly reduced triglycerides and cholesterol levels but had no impact on AAA progression. Fenofibrate therapy is unlikely to benefit patients with small AAA
Wanhainen et al	2020	The effect of ticagrelor on growth of small abdominal aortic aneurysms—a randomized controlled trial (TicAAA)	Imaging: MRI-measured AAA volume and diameter, ultrasound-measured AAA diameter. Biomarkers: Intraluminal thrombus (ILT) volume	Primary: Mean AAA volume growth (%) at 12 months (MRI). Secondary: AAA diameter growth (MRI and ultrasound), ILT volume enlargement, need for surgery, aneurysm rupture	Therapeutic trial (ticagrelor vs. placebo for reducing AAA growth)	144 randomized patients (ticagrelor *n* = 72, placebo *n* = 72)	Randomized, placebo-controlled, double-blind, multicenter trial	Ticagrelor showed no significant reduction in AAA growth compared with placebo (volume increase 9.1% vs. 7.5%, p = 0.205). Secondary outcomes (AAA diameter growth, ILT volume) also showed no significant differences. Higher rates of adverse events (especially bleeding and dyspnea) were noted in the ticagrelor group. Concluded that platelet inhibition with ticagrelor does not slow AAA progression
Golledge et al	2020	Efficacy of Telmisartan to Slow Growth of Small Abdominal Aortic Aneurysms: A Randomized Clinical Trial (TEDY)	Imaging: Ultrasound and CT measurements of AAA diameter and volume. Biomarkers: Serum CRP	Primary: AAA growth rate measured by ultrasound. Secondary: BP reduction, AAA-related events (AAA repair or mortality), health-related quality of life	Therapeutic trial (telmisartan vs. placebo for reducing AAA growth)	210 randomized (telmisartan *n* = 107, placebo *n* = 103)	Randomized, placebo-controlled trial (multicenter, double-blind)	- No significant effect of telmisartan on AAA growth rates or AAA-related events. Significant reduction in BP, but no influence on AAA progression. Increased incidence of hypotensive symptoms with telmisartan. Underpowered study due to lower than planned recruitment
Golledge et al	2021	Protocol for the Metformin Aneurysm Trial (MAT): a placebo-controlled randomised trial testing whether metformin reduces the risk of serious complications of abdominal aortic aneurysm	Imaging: Ultrasound (AAA diameter), CT/MRI if available; Biomarkers: Optional sub-study includes D-dimer, MMPs, osteoprotegerin, osteopontin	Primary: Composite endpoint of AAA-related mortality or surgical repair (“AAA events”); Secondary: AAA growth, MACE, quality of life, need for vascular surgery, all-cause mortality	Therapeutic trial (metformin vs. placebo for AAA outcomes)	Planned *n* = 1954 (977 per group); event-driven design requiring 616 primary outcome events	Randomized, placebo-controlled, double-blind, international multicenter trial	MAT is the largest trial to assess whether metformin reduces clinically important AAA events in patients without diabetes with AAA ≥ 35 mm. Unlike previous trials focused only on growth, MAT targets rupture/surgery outcomes. A 6-week run-in phase ensures metformin tolerance. Secondary outcomes include AAA growth, MACE, QoL, vascular procedures. Optional biomarker sub-study explores mechanisms. Trial completion expected in 2025. Results could identify metformin as the first effective AAA drug therapy

Trials with other agents such as fenofibrate, mast cell stabilizers [86, 90], and ticagrelor [23] have also failed to show significant efficacy. Although propranolol effectively lowers blood pressure, it is poorly tolerated and has minimal impact on aneurysm progression [91].

However, observational studies and meta-analyses have indicated potential benefits of repurposed drugs. Statins have been associated with reduced aneurysm growth, rupture risk, and perioperative mortality [8], whereas aspirin may reduce progression, particularly in nonsmokers [82].

Although numerous pharmacological interventions have been investigated, no treatment has proven sufficiently effective for standard use, highlighting the continued need for innovative and targeted approaches (Table 1) [5, 7, 9, 12, 33, 63, 77, 82, 92, 93].

## Discussion

### Pathomechanism of AAA

Although numerous studies have addressed the molecular and cellular features of AAA, our current understanding remains insufficient for defining clear therapeutic strategies. Although endothelial dysfunction, inflammation, and mechanical stress are known contributors, the temporal and causal hierarchies of these events remain unclear. Whether VSMC loss precedes ECM breakdown or whether chronic inflammation is a primary driver or a secondary phenomenon resulting from medial degeneration remains unknown. [43, 94]

The lack of a unified pathogenic model has complicated the development of targeted therapies and biomarker-guided interventions. Moreover, inter-individual variability in genetic, hemodynamic, and metabolic influences hinders the identification of universal targets [58]. These uncertainties highlight the need for integrative approaches that combine molecular profiling, imaging, and clinical phenotyping to improve patient stratification and guide personalized interventions. Consequently, clinical trials have largely failed to translate preclinical anti-inflammatory strategies into consistent outcomes as demonstrated by inconclusive results from studies investigating doxycycline and statins. [8, 30, 89, 93]

Another key limitation is the tendency to examine (inflammatory) pathways in isolation rather than their interaction with other pathophysiological mechanisms such as oxidative stress, mitochondrial dysfunction, or immune-metabolic crosstalk [95]. Despite emerging evidence regarding the roles of platelets, PVAT dysfunction, and the RAS, these pathways remain underexplored in clinical settings. Furthermore, clinical trials often treat AAA as a single, uniform disease entity, overlooking non-inflammatory contributors such as vascular biomechanics, metabolic disturbances, and impaired tissue oxygenation [96, 97].

Significant limitations remain in our understanding of how mechanisms such as microRNAs, histone modifications, and chromatin remodeling dynamically influence the SMC phenotype in AAA. Although numerous experimental studies have identified specific epigenetic regulators involved in VSMC differentiation and apoptosis, their temporal activities and interactions with inflammatory or biomechanical signals remain largely unexplored [9, 51, 98]. Similarly, although several miRNAs, including miR-26a and miR-143/145, have been implicated in AAA pathophysiology, their coordinated roles across different disease stages remain undefined [99]. Most of the existing data are derived from animal models or static human tissue samples, offering limited insights into the dynamic regulation of these molecules during aneurysm progression in real life.

Although ECM degradation is a central feature of AAA, clinical tools for assessing the dynamic remodeling of matrix components in vivo are lacking. Furthermore, although MMPs and ECM cross-linking enzymes such as lysyl oxidase are known mediators of cell wall instability, attempts to modify ECM turnover pharmacologically have not yielded durable therapeutic results [95].

While computational and imaging studies support the importance of wall shear stress, pulse pressure, and oscillatory shear index in AAA expansion, these biomechanical parameters are rarely incorporated into clinical decision-making. In addition, the complex interactions between hemodynamic stress and biological responses, such as inflammation and ECM degradation, remain poorly understood. [58, 66]

The role of clonal hematopoiesis of indeterminate potential (CHIP) in vascular inflammation is gaining increasing attention. Although several studies have suggested a strong association between CHIP-related mutations and vascular disease, it remains unclear whether CHIP is a direct causal factor in aneurysm development or simply a correlated marker of risk [100]. In addition, most clinical trials, including those evaluating doxycycline, statins, and antiplatelet therapies, do not stratify patients based on these profiles, thus limiting personalized treatment strategies [23, 25, 30].

### Diagnostics and screening of AAA

Despite the high-level evidence, AAA screening has not been consistently implemented in clinical practice. A major issue is the declining prevalence of AAA, particularly in countries with strong public health measures and low smoking rates. For instance, the prevalence in 65-year-old Swedish men is less than 1.5%, making universal screening less cost-effective [101]. Countries such as Finland, which lack national AAA screening programs, also cite concerns over cost, resource allocation, and diminishing returns as prevalence declines. By contrast, Sweden’s established program has achieved lower rupture rates and more elective repairs, supporting the long-term value of organized population screening [22, 29–33, 102].

Another barrier is limited patient participation. In the MASS and Western Australia trials, many AAA-related deaths occurred among individuals who declined screening or were later deemed unfit for surgery [13, 16]. These findings highlights the fact that screening efficacy depends on patient engagement and operability, which are often compromised in older or medically complex individuals. Methodological inconsistencies contribute to challenges in AAA research. Diagnostic imaging methods vary widely, from standard ultrasonography to advanced techniques such as CTA and CMR. These discrepancies lead to variations in aneurysm size, growth rate estimation, and risk stratification. Furthermore, differences in follow-up intervals and study durations may obscure long-term outcomes or delay treatment effects. [6, 13, 14, 21] Schäberle noted that the lack of standardized measurement techniques may significantly influence clinical decisions, underscoring the need for strict imaging protocols [70].

Limited evidence exists, especially in women with a lower prevalence of AAA, but a higher risk of rupture at smaller diameters. Nevertheless, routine screening of women is not recommended because of insufficient data and concerns regarding overdiagnosis [14].

Although developing targeted screening strategies based on individualized risk profiles shows promise, such approaches have yet to be widely implemented. A systematic review by Musto et al. identified 37 risk prediction models; however, most lacked external validation and integration into electronic health records, limiting their clinical applicability [5].

Several technological innovations have emerged to refine the diagnosis and monitoring of AAA. Among them, artificial intelligence (AI) and machine learning (ML) models have been investigated for their potential to enhance the interpretation of imaging data and automate the prediction of aneurysm growth. Abbas et al. demonstrated that AI-based predictive models could effectively integrate clinical, morphological, and biomechanical imaging features to forecast aneurysm progression, thereby supporting individualized surveillance strategies and determining the optimal timing for intervention [103]. Despite their promise, the clinical applications of AI and ML in AAA management remain limited. The same research group identified several key challenges including the heterogeneity of imaging data, lack of transparency in model decision-making, "black-box" models, and insufficient external validation in diverse clinical settings. Ethical and legal concerns, particularly regarding data privacy and liability, further complicate implementation. These limitations underscore the need for standardized, interpretable, and rigorously validated AI systems before these tools can be reliably integrated into personalized AAA care [102].

In addition to imaging, biomarkers are gaining attention as potential tools for risk stratification, particularly in patients under surveillance with small AAA. Kuivaniemi et al. and Sakalihasan et al. outlined the genetic and inflammatory profiles associated with aneurysm development. However, their translation into clinical applications remains limited [104, 105].

A more recent study by Golledge et al. investigated four serum biomarkers (C-reactive protein [CRP], fibrinogen, neutrophil-to-lymphocyte ratio, and homocysteine) in 471 patients with small AAAs (30–54 mm). Their prospective cohort analysis demonstrated that elevated CRP levels were independently associated with an increased risk of major adverse cardiovascular events, including myocardial infarction, stroke, and cardiovascular death. After adjusting for traditional risk factors, CRP level remained a significant predictor and improved risk classification metrics, suggesting that it may be a relevant adjunct for clinical decision-making [106].

A recent scoping review by Khan et al. identified over 45 protein biomarkers with prognostic potential in AAA, spanning inflammation, tissue remodeling, cardiovascular function, and hemostasis. Notable markers include B-type natriuretic peptide, lipoprotein-associated phospholipase A2, citrullinated histone H3, and elastase, all of which are associated with the presence or progression of aneurysms. Although many candidates lack external validation, this review highlights the growing trend towards multi-marker strategies to enhance AAA risk prediction and personalized monitoring approaches. [107]

### Current limitations in AAA management

The clinical management of AAAs continues to face considerable challenges due to an incomplete understanding of their pathogenesis and progression. Despite decades of intensive research, pharmacological interventions have not demonstrated consistent efficacy in slowing aneurysm growth or preventing aneurysm rupture. Notably, trials like TEDY and AARDVARK, which assessed angiotensin-converting enzyme inhibitors and doxycycline, respectively, were limited by low statistical power and heterogeneous inclusion criteria. These limitations have constrained the development of robust and generalizable conclusions regarding potential pharmacotherapies for AAA. [1, 26, 27, 30, 80, 83–89]

Early studies suggested that doxycycline reduces MMP-9 levels [108]; however, these findings were not confirmed in subsequent trials such as PHAST [30]. Similarly, observational data have indicated the possible benefits of statins and metformin in reducing AAA expansion and rupture risk; however, these results require validation in ongoing randomized controlled trials, including the Metformin Aneurysm Trial and the Limiting AAA with metformin (LIMIT) trial (Table 1) [77, 92].

A significant obstacle in AAA pharmacological research is the marked variability in trial designs. Differences in the inclusion criteria, such as the size range of aneurysms included or baseline patient risk, hinder comparability between studies. For instance, the TEDY focused exclusively on small aneurysms, whereas the AARDVARK adopted a broad range of inclusion criteria. Inconsistencies in patient demographics, including age, sex, and comorbidities, further complicate the interpretation of study outcomes, because these factors can significantly influence disease progression. [1, 80, 81]

Furthermore, the definitions of trial endpoints varied considerably. Some studies prioritized the aneurysm growth rate as the primary outcome, while others focused on clinical endpoints such as rupture or all-cause mortality. Confounding factors such as lifestyle and environmental influences, including smoking, physical activity, and dietary habits, are often inconsistently reported or controlled. This lack of standardization limited our ability to perform pooled analyses and draw clinically meaningful conclusions. [8, 9, 11, 16, 23, 27, 32, 80, 81, 84, 86, 89, 93]

## Future directions and implications for research

### Pathomechanisms, translational perspectives and imaging in AAA research

Contemporary research aims to detect patients at high risk of rapid aneurysm progression and rupture early rather than relying solely on surgical thresholds. This requires multi-parametric tools, including demographic profiling, imaging biomarkers, and genetic markers. Modern imaging modalities play a central role in the framework.

Multiomics analyses have begun to clarify the molecular pathways underlying inflammation, smooth muscle loss, and matrix degradation. However, these insights require better integration with patient stratification data for clinical application [109].

Computational modeling and AI are increasingly used to link biomechanical forces with molecular phenotypes, thereby enabling precision medicine strategies [25, 103]. Consortia such as the NIH Complement-ARIE promote non-animal methodologies (NAMs), including microphysiological systems and in silico vascular models, to reduce animal dependence and enhance translational potential [110]. The DFG-funded TRR 256 consortium in Germany is advancing the knowledge of aortic structure and function using integrated models and human tissue analysis [22], while the VASCUNET registry supports clinical correlation through population-level surveillance and promotes quality improvement by benchmarking vascular surgical outcomes across countries [111].

Magnetic resonance angiography (MRA) techniques, such as four-dimensional (4D) Flow MRI, allow time-resolved assessment of blood flow and calculation of wall shear stress (WSS), oscillatory shear index (OSI), and relative residence time (RRT). These parameters correlate with endothelial dysfunction and thrombus formation [112, 113]. MR elastography (MRE) measures wall stiffness, with increased values linked to structural wall weakness [114]. Pulse wave velocity (PWV), another stiffness metric, has a prognostic value post-EVAR [69]. These measurements may play important roles in future diagnostics.

**Fig. 2:**
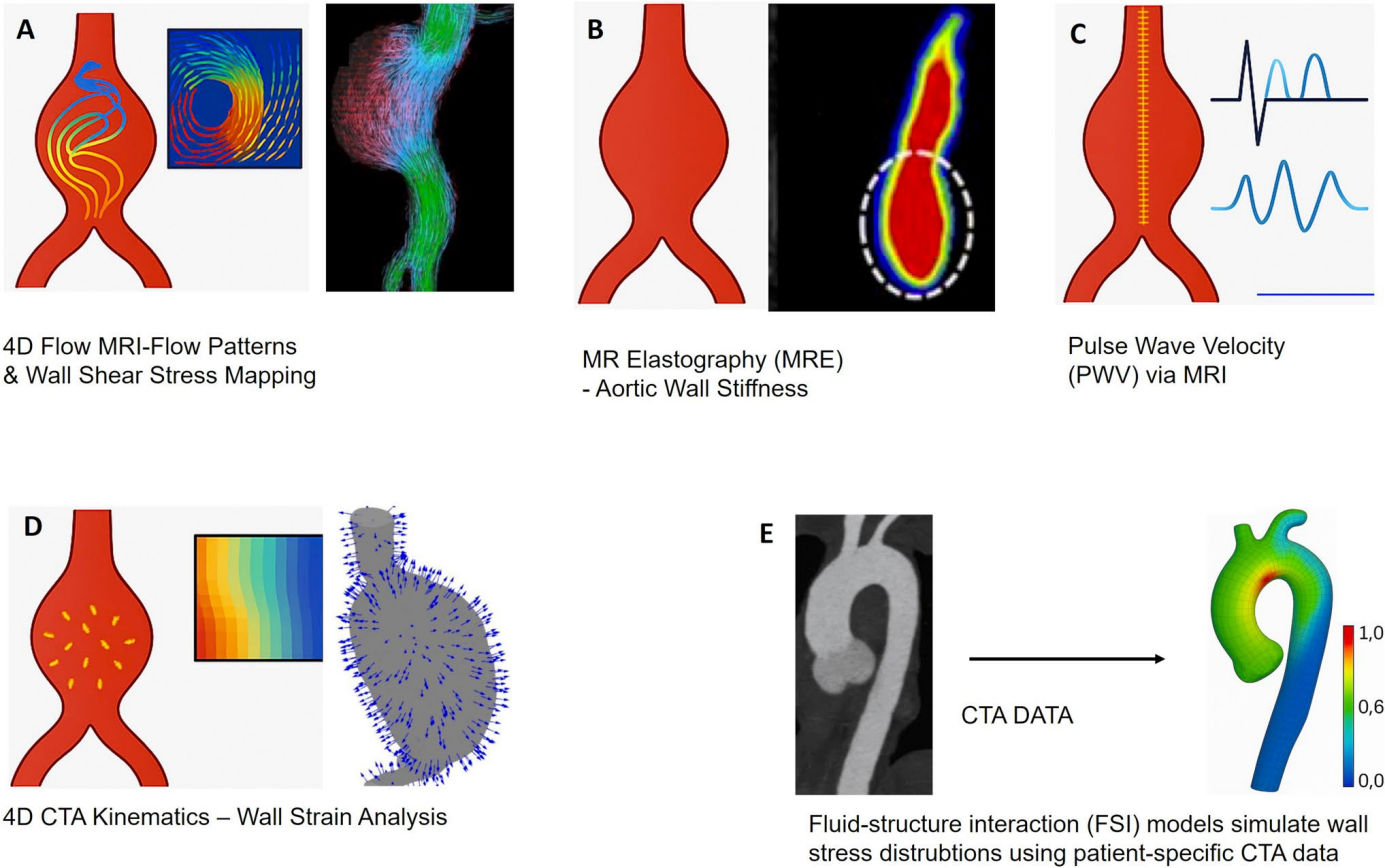
Advanced imaging techniques for AAA assessment (A) 4D Flow MRI visualizing blood flow and wall shear stress. (B) MR elastography mapping regional aortic wall stiffness. (C) Pulse wave velocity measurement reflecting arterial elasticity. (D) 4D CTA strain analysis showing aortic wall deformation. (E) Fluid–structure interaction (FSI) models simulate wall stress distributions using patient-specific CTA data

CTA remains the gold standard for anatomical assessment but is increasingly being used to evaluate dynamic aortic wall behavior. 4D CTA combined with image registration enables strain mapping and reveals regions of reduced circumferential strain in the AAA [115, 116]. Fluid–structure interaction (FSI) models simulate wall stress distributions using patient-specific CTA data, thereby improving the prediction of rupture-prone zones (Fig. 2) [117].

AI-enhanced approaches further refine biomechanical modeling, with machine learning algorithms predicting rupture risk more accurately than diameter alone [118]. Multimodal studies seek to integrate imaging features, biomarkers, and clinical parameters into predictive frameworks. The integration of refined preclinical models, AI-supported analytics, and advanced imaging modalities is a comprehensive strategy for AAA research. Moving from static measurements to mechanistically informed patient-specific assessments enhances early detection, risk stratification, and preventive vascular care.

### Current and future pharmacotherapies targeting AAA pathogenesis

Despite the lack of approved pharmacological treatments for AAA, several promising strategies are currently being pursued to improve disease management.

Metformin has emerged as a particularly promising candidate. A large-scale trial, the Metformin Aneurysm Trial, is currently ongoing [77], and a 2024 meta-analysis by Kristensen et al. suggested a statistically significant reduction in AAA growth (− 0.73 mm/year) in patients receiving metformin, although the overall quality of evidence was rated as low [83, 119].

A recent Mendelian randomization and colocalization study by Liu et al. investigated the causal relationship between metformin use and the risk of aortic aneurysms, with a particular focus on abdominal aortic aneurysms. Utilizing large-scale genomic data from the UK Biobank and FinnGen cohorts, the authors demonstrated that metformin use is significantly associated with a reduced risk of AA (OR ≈ 0.005, 95% CI: 7.30 × 10⁻.^5^–0.33, p = 0.01). Multivariate Mendelian randomization further indicated that the previously reported protective effect of type 2 diabetes on AA was primarily attributable to metformin treatment rather than to the diabetic state itself. They found that increased expression of NDUFA6 (NADH: ubiquinone oxidoreductase subunit A6), a component of the mitochondrial complex I, and CYB5B (cytochrome b5 type B) were associated with a higher risk of AA. Colocalization analyses supported a shared genetic basis between AA risk and the expression of these genes, particularly NDUFA6, in vascular tissues such as the aorta and coronary and tibial arteries. These findings suggest that metformin may exert protective effects by modulating mitochondrial function and immune pathways. [120]

Similarly, statins are being investigated for their pleiotropic effects, particularly their potential to modulate vascular inflammation and extracellular matrix remodeling, beyond their lipid-lowering properties [121].

**Fig. 3:**
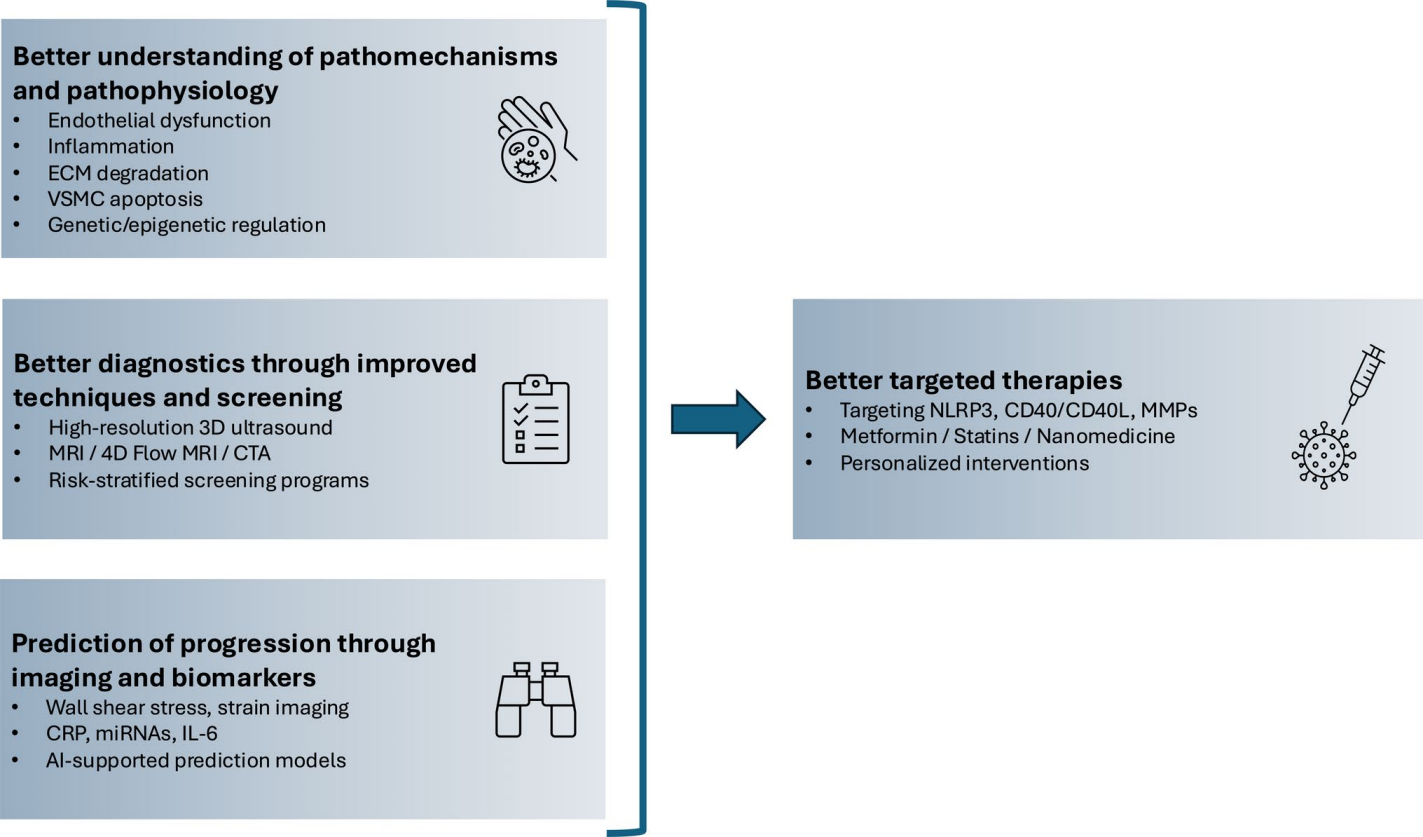
Future perspectives in abdominal aortic aneurysm (AAA) management, highlighting advancements in understanding disease mechanisms, improving diagnostics and prediction via imaging and biomarkers, developing targeted therapies, and implementing personalized interventions. AI: artificial intelligence; CD40/CD40L: Cluster of Differentiation 40 and its ligand; CTA: computed tomography angiography; ECM: extracellular matrix; IL-6: interleukin 6; miRNAs: Micro ribonucleic acid; MMPs: Matrix metalloproteinases; MRI: magnetic resonance imaging; NLRP3: NOD-like receptor protein 3; VSMC: vascular smooth muscle cells

Beyond conventional pharmacotherapy, future therapeutic strategies are expected to target inflammatory and immunological pathways that play central roles in AAA pathogenesis. Key candidates include the CD40/CD40L-TRAF6 axis, myeloperoxidase, toll-like receptor (TLRs), and NOD-like receptor P3 (NLRP3) inflammasome, which have shown consistent protective effects in preclinical models by suppressing vascular inflammation and ECM degradation. For example, the inhibition of CD40-TRAF6 signaling attenuated vascular inflammation and AAA formation in murine models [122]. Myeloperoxidase deficiency or pharmacological inhibition reduces aneurysm progression in mice, likely by lowering oxidative stress [123]. Similarly, the genetic deletion or blockade of TLR4 and NLRP3 results in significantly smaller aneurysms and decreased inflammatory responses [124, 125]. Although these insights have yet to be translated into clinical trials, they offer a strong rationale for future disease-modifying therapies. As illustrated in Fig. 3, future improvements in AAA management are expected through a better understanding of the underlying pathophysiological mechanisms (e.g., inflammation and ECM degradation), enhanced diagnostics enabled by advanced imaging and screening technologies, more accurate prediction of disease progression using biomarkers and imaging, and the development of targeted therapies based on newly identified mechanisms.

Despite considerable advances in our understanding of the molecular and biomechanical mechanisms underlying AAA progression, clinical translation remains challenging. To address this gap, large and diverse multicenter registries such as the TRR/AAA Progression Registry are expected to develop validated risk prediction models and enable personalized therapeutic strategies, thereby helping close the translational gap in AAA research [22, 26]. Importantly, the registry was designed to integrate genetic, immunological, cellular, and biomechanical parameters to create a comprehensive dataset reflecting the biological complexity of AAA. By enrolling a large and diverse multicenter cohort and applying multimodal longitudinal assessment strategies, this initiative seeks to develop a validated risk score for aneurysm growth and adverse outcomes, and to identify subgroups that might benefit from early, targeted interventions.

Unlike earlier observational studies or size-only surveillance protocols, TRR 259 combines standardized clinical data collection, serial high-resolution imaging, and translational endpoints that align directly with mechanistic hypotheses. It has been hypothesized that patients with rapidly progressing AAAs exhibit distinct immunometabolic profiles and biomechanical signatures that are detectable years before conventional size thresholds are reached. By identifying these features early, future trials will be able to molecularly stratify patients and test disease-modifying therapies in targeted subgroups that are most likely to benefit [22, 26].

## Conclusion

AAA is a silent but serious disease with limited treatment options. Future studies should clarify whether early molecular and imaging profiles can distinguish stable from fast-growing aneurysms, thereby enabling earlier and more personalized care.

## Data Availability

The data that support the findings of this review are available from the corresponding author upon reasonable request.
